# A Validated Stability-Indicating Liquid-Chromatographic Method for Ranitidine Hydrochloride in Liquid Oral Dosage Form

**DOI:** 10.3797/scipharm.1101-06

**Published:** 2011-02-12

**Authors:** Nitish Sharma, Surendra Singh Rao, Namala Durga Atchuta Kumar, Pingili Sunil Reddy, Annarapu Malleswara Reddy

**Affiliations:** 1 Analytical Research and Development, IPDO, Dr. Reddy’s Laboratories Ltd. Bachupally, Hyderabad-500072, A.P, India; 2 Department of chemistry, J. N. T. University, Kukatpally, Hyderabad-85, A.P, India

**Keywords:** Method development, Stability-indicating, Ranitidine, Stress conditions, Impurities, Oral Solution, RP-HPLC

## Abstract

A selective, specific and stability-indicating gradient reverse phase high-performance liquid chromatographic (HPLC) method was developed for the determination of Ranitidine in presence of its impurities, forced degradation products and placebo substances such as saccharide and parabens. Ultraviolet detection was performed at 230 nm. Separate portions of the drug product and ingredients were exposed to stress conditions to induce oxidative, acidic, basic, hydrolytic, thermal and photolytic degradation. Ranitidine was found to degrade significantly at acidic, basic and oxidative stress conditions but was stable at heat and humidity. The developed method was validated as per International Conference on Harmonization (ICH) guidelines. The method was validated over this range for (i) system suitability (ii) specificity, (iii) precision, (iv) limit of detection and limit of quantification, (v) linearity, (vi) accuracy, (vii) robustness. The method was found to be precise, accurate, linear and robust. The proposed method was successfully employed for estimation of Ranitidine impurities in pharmaceutical preparations.

## Introduction

**Fig. 1.**
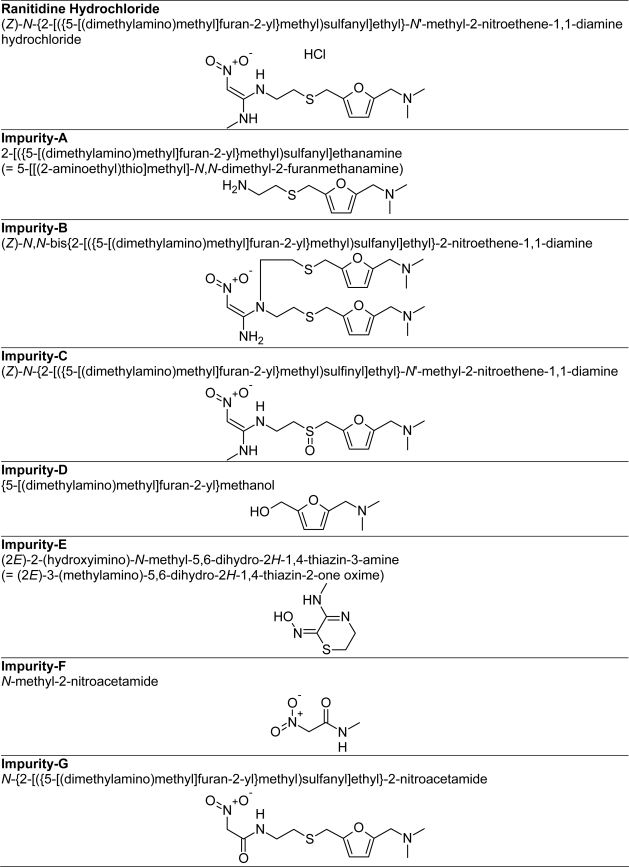
Chemical structure of Ranitidine HCl and its officinal impurities (USP 31)

Ranitidine hydrochloride (HCl), USP, is a histamine H2-receptor antagonist. Chemically it is (*Z*)-*N*-{2-[({5-[(dimethylamino)methyl]furan-2-yl}methyl)sulfanyl]ethyl}-*N*′-methyl-2-nitroethene-1,1-diamine hydrochloride (Fig. 1).

Many analytical approaches are available for the related substances of ranitidine in tablet formulations and drug substances [1–3]. A number of assay methods for determination of ranitidine in pharmaceutical formulations and human plasma is also available [4–6]. However, all the above-mentioned methods are orientated to the determination of the active pharmaceutical compound. Now-a-days, pharmaceutical industry is forced to assess a strict control of impurities when manufacturing drug substance and drug products. Determination of impurities during the development of separation methods is one of the main and difficult tasks for pharmaceutical analysts, especially if more and more impurities of closely related structure require determination. Methods are available for estimation of related substance in ranitidine with capillary electrophoresis [7, 8].

To the best of our knowledge, none of the currently available analytical methods can separate all the known related compounds and degradation impurities of ranitidine hydrochloride dosage form. Attempts were made to develop a stability indicating LC method for estimation of related substance of ranitidine in liquid orals (syrups). The published Ranitidine impurity method demonstrates analysis of estimation of Ranitidine impurities in presence of placebo like saccharide and parabens with detection wavelength at 230 nm. This paper deals with the forced degradation of ranitidine syrup under stress condition like acid hydrolysis, base hydrolysis, oxidation, heat and UV light. This paper also deals with the validation of the developed method for the accurate quantification of impurities of ranitidine.

## Experimental

The empirical formula of Ranitidine Hydrochloride is C_13_H_22_N_4_O_3_S•HCl, representing a molecular weight of 350.87. 1 mL of ranitidine Syrup contains 16.8 mg of ranitidine HCl equivalent to 15 mg of ranitidine.

### Chemicals and reagents

Syrup and standards of ranitidine and its seven impurities namely imp-A, imp-B, imp-C, imp-D, imp-E, imp-F and imp-G (Fig. 1) were supplied by Dr. Reddy’s laboratories limited, Hyderabad, India. The HPLC grade acetonitrile, and analytical grade KH_2_PO_4_ and ortho phosphoric acid were from Merck, Darmstadt, Germany. Water was purified by a Millipore (Bedford, MA, USA) Milli-Q water-purification system and passed through a 0.22 μm membrane filter (Durapore; Millipore, Dublin, Ireland) before use.

### Equipment

The waters HPLC PDA 2996 system used consists of a Quaternary solvent manager, a sample manager and a Photodiode array UV detector. The output signal was monitored and processed using empower software. Water baths equipped with MV controller (Julabo, Seelbach, Germany) were used for hydrolysis studies. Photo stability studies were performed in a photo stability chamber, UV light (200 watt hours / square meter), sun light (1.2 Million Lux hours) Calibrated (Sanyo, Leicestershire, UK). Thermal stability studies were performed in a dry air oven (MACK Pharmatech, Hyderabad, India).

### Chromatographic Conditions

The analytes were separated on (100 × 4.6 mm, 3 μm) ACE C18 column with mobile phase containing a gradient mixture of solvent A and B at column oven temperature of 40^o^C with a gradient run program at a flow-rate of 1.0 mL min^−1^. 0.05 M potassium dihydrogen orthophosphate buffer, pH adjusted to 6.5 with orthophosphoric acid was used as buffer. Buffer and acetonitrile in 98:2 v/v ratios was used as solvent A and MilliQ water and acetonitrile in 5:95 v/v ratio was used as solvent B. The separation was achieved by gradient elution (T/%B) set as 0/0, 10/5, 25/15, 35/20, 40/55, 55/0 and 60/0. The mobile phase was filtered through a nylon membrane (pore size 0.45 μm) and degassed with a helium spurge for 10 min, before use. UV detection was performed at 230 nm. The sample injection volume was 40 μl.

### Preparation of Stock Solutions

A standard stock solution (Stock A) of ranitidine (0.65 mg/mL) prepared by dissolving 72 mg Ranitidine hydrochloride of reference standard in 100 mL solvent-A. Working solutions of 32.5 μg/mL (Stock-B) diluting stock-A 5.0 mL to 50 mL with solvent-A and 1.625 μg/mL (Stock-C) diluting stock-B 5.0 mL to 100 mL with solvent-A were prepared for the related substance determination.

The impurity stock solution was prepared by dissolving an accurately weighed amount of impurity A, imp-B, imp-C, imp-D, imp-E, imp-F and imp-G in Solvent-A, resulting in a concentration of 0.088 mg/mL (Stock-D) of (2.2 mg each impurity in 25 mL volumetric flask) of each impurity.

System suitability solution containing (0.33 mg/mL) 30 mg Ranitidine and 1.76 μg/mL (2 mL of Stock-D) each impurity was also prepared.

### Preparation of Sample Solution

2 mL ranitidine syrup (equivalent to 30 mg ranitidine and 33.3mg ranitidine HCl.) was transferred to a 100 mL volumetric flask, 50 mL solvent A was added. The mixture was then sonicated for 10 minute and diluted to volume to give a solution containing 300 μg/mL. The above solution was centrifuged at 4000rpm for 10 minutes in order to eliminate insoluble excipients. This solution was filtered through a 0.45 μm pore size Nylon 66 membrane filter and inject in HPLC system as per chromatographic conditions mention in section 2.3.

### Method Validation

The proposed method was validated as per ICH guidelines [9].

#### Specificity

Specificity is the ability of the method to measure the analyte response in the presence of its potential impurities. A study was conducted to demonstrate the effective separation of Ranitidine Hydrochloride and its impurities. Also study was intended to ensure the effective separation of degradation peaks of formulation ingredients at the retention time of Ranitidine Hydrochloride and its impurities. Separate portions of drug product and ingredients were exposed to following stress conditions to induce degradation.

The drug product was subjected to base hydrolysis using 0.1 N Sodium hydroxide, acid hydrolysis with 0.1N Hydrochloric acid for duration of 30 minute. Oxidation study was performed with 0.1 % Hydrogen Peroxide solution at room temperature for 30 minute. On photo stability study, drug product was sufficiently spread on Petri plates (1 mm thick layer), exposed to sunlight and UV light (200 watt hours / square meter), sun light (1.2 Million Lux hours) at ambient conditions for 10 days. Humidity study was performed separately by exposing the drug product to humidity at 25°C, 90% RH for 7 days. Thermal degradation study was performed by heating drug product at 60° C for 10 days. Similarly placebo samples were prepared as like as drug product by exposing formulation matrices without drug substance.

Peak purity test was carried out for the ranitidine peak by using PDA detector in stress samples.

#### Precision

The precision of the related substance method was checked by injecting six individual preparations of ranitidine (0.3 mg/mL) spiked with 0.50% of imp-A, imp-B, imp-C, imp-D, imp-E, imp-F and imp-G with respect to ranitidine analyte concentration. % R.S.D. of area for each imp-A, imp-B, imp-C, imp-D, imp-E, imp-F and imp-G was calculated.

The intermediate precision of the method was also evaluated using different analyst and different instrument in the same laboratory.

#### Limits of Detection (LOD) and Quantification (LOQ)

The LOD and LOQ for imp-A, imp-B, imp-C, imp-D, imp-E, imp-F and imp-G were determined at a signal-to-noise ratio of 3:1 and 10:1, respectively, by injecting a series of dilute solutions with known concentrations. LOD and LOQ were experimentally verified by injecting six replicate injection of each impurity at the concentration obtained from above values.

#### Linearity

A series of solutions of Ranitidine impurities in the concentration ranging from limit of quantification level to 300% of standard concentration were prepared and injected into the HPLC system. Correlation coefficient Value for the slope and Y-intercept of the calibration curve was calculated.

#### Accuracy

To confirm the accuracy of the proposed method, recovery studies were carried out by standard addition technique. The accuracy study of impurities were carried out in triplicate at LOQ, 50%, 100%, 150%, 200% and 300% of the target concentration level 0.5% of ranitidine analyte concentration (300 μg/mL). The percentages of recoveries for impurities were calculated.

#### Robustness

To determine the robustness of the developed method, experimental conditions were deliberately altered and the resolution between ranitidine imp-A, imp-B, imp-C, imp-D, imp-E, imp-F and imp-G was recorded. The flow rate of the mobile phase was 1.0 mL/min. To study the effect of flow rate on the resolution, flow was changed by 0.2 units from 0.8 to 1.2 mL/min. The effect of the column temperature on resolution was studied at 35° and 43° C instead of 40° C. The effect of the percent organic strength on resolution was studied by varying acetonitrile by −10% +10%. While other mobile phase components was held constant as stated in Section 2.3.

#### Solution Stability and Mobile Phase Stability

The solution stability of ranitidine and its impurities in the related substance method was carried out by leaving spiked sample solutions in tightly capped volumetric flasks at room temperature for 48 hours. Content of ranitidine imp-A, imp-B, imp-C, imp-D, imp-E, imp-F and imp-G were determined for every 24 hours interval up to the study period. The mobile phase stability was also carried out for 48 hours by injecting the freshly prepared sample solutions for every 24 hours interval. Content of ranitidine imp-A, imp-B, imp-C, imp-D, imp-E, imp-F and imp-G were checked in the test.

## Results and Discussion

### Method Development and Optimization

The main criteria for development of a successful HPLC method for determination of Ranitidine HCl and its related Substances in oral solution were: the method should be able to determine all substances in a single run and should be accurate, reproducible, robust, stability indicating, free of interference from blank / placebo / impurities / degradation products and straightforward enough for routine use in quality control laboratory.

**Fig. 2.**
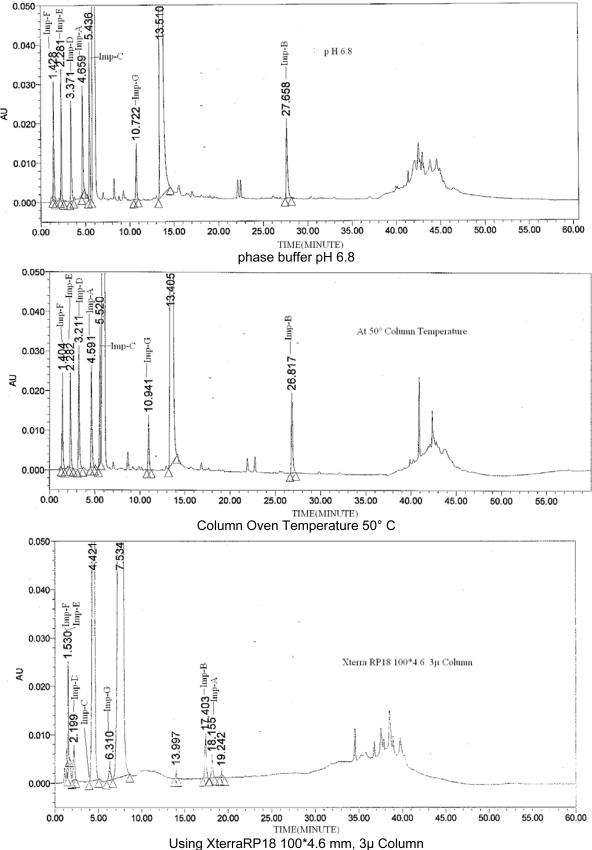
Typical chromatograms of ranitidine from method development trails

**Fig. 3.**
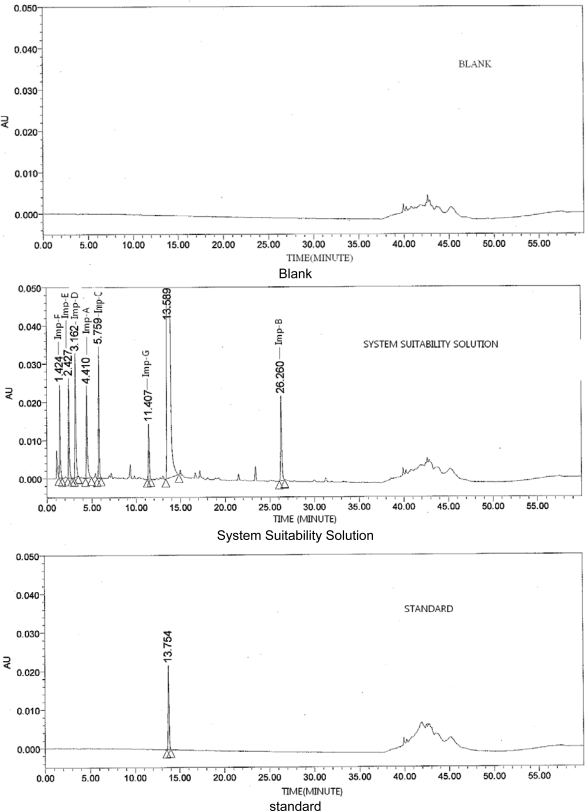

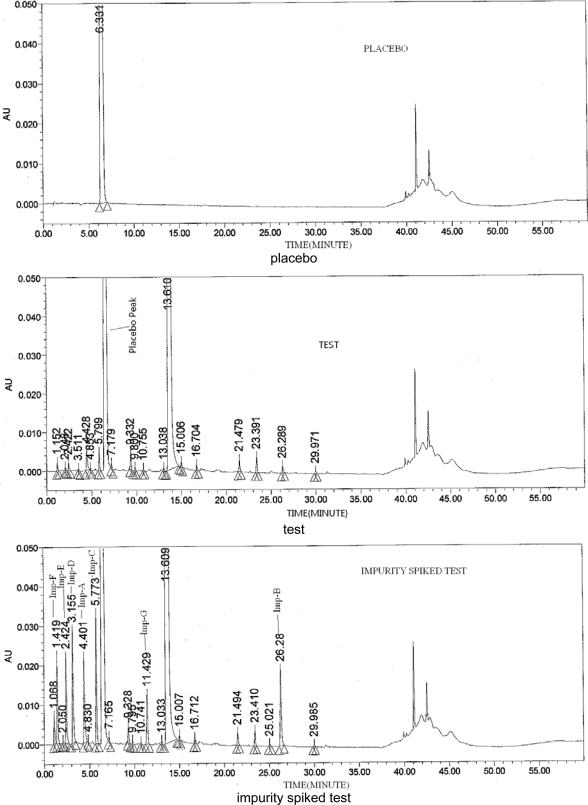
Typical chromatograms of Ranitidine Oral Solution at optimized chromatographic conditions

The main objective of the chromatographic method was to achieve good resolution between critical closely eluting pair ranitidine impurity-D and impurity-E, to well resolve impurity-C peak from placebo peak, and to find Symmetrical peak shape of Ranitidine Hydrochloride and its Impurities. To elute parabens peaks, a gradient elution with 95 % acetonitrile in the solvent B has been selected. In order to achieve symmetrical peak of Ranitidine Hydrochloride and its Impurity and more resolution between impurity-D and impurity-E, stationary phases like C18 (different brand), ACE C18 250 × 4.6, 5μ column, Xterra RP 18 100 × 4.6 mm, 5μ, Xterra RP18 100 × 4.6, 3.5μ (Fig. 2) were studied but good resolution has achieved with ACE C18 100 × 4.6, 3μ column (Fig. 3). The flow rate of 1.0 mL/min was selected with regards to the backpressure and analysis time as well. Column oven temperature is also studied (50°C and 40°C) and found that 40°C temperature is more appropriate with respect to resolution and peak shape (Fig. 2).

Attempts were also made with gradient elution with solvents A and B (Section 2.3) using pH 6.8 buffer conditions, the resolution between impurity C and placebo peak was decreased (Fig. 2).

It was found that use of buffer prepared by adjusting the pH of 0.05M potassium dihydrogen phosphate to 6.5 (PKa of Ranitidine is 8.2) with orthophosphoric acid (solvent A: buffer, acetonitrile in the ratio 98:2, v/v and solvent B: Milli Q water, acetonitrile in the ratio 5:95, v/v) with column temperature was maintained at 40° C and gradient elution (T/%B) was set as 0/0, 10/5, 25/15, 35/20, 40/55, 55/0 and 60/0 gives enabled separation for all pair compounds and eluted Ranitidine as a symmetrical peak (Fig. 3 and table 1). No interfering peaks were observed in blank & placebo, indicating that signal suppression or enhancement by the product matrices was negligible (Fig. 3).

The empirical formula of Ranitidine Hydrochloride is C_13_H_22_N_4_O_3_S•HCl, representing a molecular weight of 350.87. Each 1 mL of ranitidine Syrup contains 16.8 mg of ranitidine HCl equivalent to 15 mg of ranitidine.

### Validation of the Method

#### System Suitability

System suitability parameters were measured so as to verify the system, method and column performance. Results of other system suitability parameters such as relative retention time of each impurity, resolution between Impurity E and Impurity D, tailing factor and similarity factor (between two standard preparations) are presented in table 1. As seen from this data, the acceptable system suitability parameters would be: relative retention time of each impurity should comparable, resolution between Impurity E and Impurity D is not less than 1.5, Tailing factor for Ranitidine peak in standard solution is not more than 2.0, similarity factor (between two standard preparations) is not less than 0.98 and not more than 1.02. Spiked chromatogram of impurity / degradation products with Ranitidine is presented in Figure 2.

#### Specificity

All forced degradation samples were analyzed at an initial concentration of Ranitidine with HPLC conditions mentioned in Section 2.3 using PDA detector to ensure the homogeneity and purity of Ranitidine peak. Significant degradation of Ranitidine was observed in oxidative (1.0% H2O2 at 60 °C for 30 minutes), Acid (0.1 N HCl at 60 °C for 30 minutes) and Base (0.1 N NaOH at 60 °C for 30 minutes) conditions leading to the formation of impurities. % Degradation has summarized in table 2.

#### Precision

The % R.S.D. for the area of ranitidine imp-A, imp-B, imp-C, imp-D, imp-E, imp-F and imp-G in related substance method precision study was within 2%, conforming good precision of the method. To check reproducibility of method Intermediate precision study has been performed in same laboratory with different day on different HPLC system and column. % RSD values presented in table 3.

#### Limits of Detection and Quantification

The determination limit of detection, limit of quantification of all the impurities namely ranitidine imp-A, imp-B, imp-C, imp-D, imp-E, imp-F and imp-G are reported in table 3. The precision at the LOQ concentrations for ranitidine imp-A, imp-B, imp-C, imp-D, imp-E, imp-F and imp-G were below 7.5 %. LOD, LOQ values presented in table-3

#### Linearity

The result shows that an excellent correlation existed between the peak area and concentration of the analyte. Linear calibration plot for the related substance method was obtained over the calibration ranges tested, i.e. LOQ to 300 % for impurity imp-A, imp-B, imp-C, imp-D, imp-E, imp-F and imp-G. The correlation coefficient obtained was greater than 0.997 (table 3). The above result show that an excellent correlation existed between the peak area and the concentration of imp-A, imp-B, imp-C, imp-D, imp-E, imp-F and imp-G.

#### Accuracy

The percentage recovery of impurities in ranitidine samples varied from 90 to 110 % at LOQ, 50%, 100%, 150%, 200% and 300 % levels of target 0.5 % level. The LC chromatogram of spiked sample at 0.5% level of all seven impurities in ranitidine oral solution is shown in figure 2. % Recovery values for impurities are presented in table 4.

#### Robustness

In all the deliberate varied chromatographic conditions (flow rate, column temperature and composition of organic solvent), the resolution between critical pairs, i.e. imp-D and imp-E was greater than 1.5, illustrating the robustness of the method.

#### Stability in Solution and in the Mobile Phase

No significant changes were observed in the content of impurities namely imp-A, imp-B, imp-C, imp-D, imp-E, imp-F and imp-G during solution stability and mobile phase stability experiments when performed using the related substance method. The solution stability and mobile phase stability experiment data confirms that the sample solutions and mobile phases used during related substance determination were stable for 48 hours.

## Conclusions

The gradient HPLC method developed for ranitidine and related substances in pharmaceutical dosage forms is precise, accurate, linear, robust, rugged and specific. Satisfactory results were obtained from validation of the method. The method is stability-indicating and can be used for routine analysis of production samples and to check the stability of samples of ranitidine.

## Figures and Tables

**Tab. 1.** System Suitability

**Compound name**	**RT**	**RRT^a^**	**Tailing factor**
IMP F	1.42	0.10	1.3
IMP-E	2.42	0.18	1.4
IMP-D	3.10	0.23	1.4
IMP-A	4.45	0.32	1.5
IMP-C	5.78	0.42	1.1
IMP-G	11.41	0.84	1.1
Ranitidine	13.75	1.00	1.1
IMP-B	27.357	1.96	1.4

Similarity factor for two standard preparations observed 0.99; Resolution between Impurity E and Impurity D observed 1.71.

a Relative retention times (RRT) were calculated against the retention time (RT) of Ranitidine.

**Tab. 2.** Specificity

**Stress Condition**	**Drug Product**							
	**%^a^**	**Imp-A**	**Imp-B**	**Imp-C**	**Imp-D**	**Imp-E**	**Imp-F**	**Imp-G**
Refluxed with 0.1N HCl solution for about 30 min at 60°C	11.3	0.59	0.01	0.14	4.64	4.78	0.11	1.18
Refluxed with 0.1N NaOH solution for about 30 min at 60°C	12.4	1.11	0.02	0.30	0.05	0.09	0.36	10.85
Refluxed with 0.1% Hydrogen peroxide for about 30 min at 60°C	7.9	0.13	0.03	6.65	0.09	0.03	NIL	1.19
Exposed to Sunlight for about 1.2 Million Lux hrs	4.5	0.54	0.03	0.99	0.01	0.03	NIL	0.07
Exposed to UV light both at shorter and longer wavelengths for about 200 watt hrs / m^2^	1.6	0.30	0.04	0.64	0.02	0.02	NIL	0.04
Dry heating done at 60°C for about 12 hrs	0.3	0.19	0.04	0.23	NIL	0.03	NIL	0.06

a % degradation.

**Tab. 3.** LOD, LOQ values, Regression and Precision Data.

**Parameter**	**Imp-A**	**Imp-B**	**Imp-C**	**Imp-D**

LOD (μg/ml)	0.02	0.01	0.01	0.01
LOQ (μg/ml)	0.08	0.05	0.03	0.04
Regression equation (*y*)				
Slope (*b*)	800058.78	110376.41	104469.9	126925.17
Intercept (*a*)	−5220.21	−5142.23	−761.31	−982.74
Correlation coefficient	0.997	0.999	0.999	0.999
Precision (%RSD)	0.1	0.4	0.4	0.1
Intermediate precision (%RSD)	0.2	0.4	0.2	0.3

**Parameter**	**Imp-E**	**Imp-F**	**Imp-G**	

LOD (μg/ml)	0.01	0.01	0.02	
LOQ (μg/ml)	0.04	0.04	0.08	
Regression equation (*y*)				
Slope (*b*)	81026.01	55442.92	58284.3	
Intercept (*a*)	−1541.70	−1746.62	−1206.81	
Correlation coefficient	0.997	0.999	0.999	
Precision (%RSD)	0.1	0.1	0.5	
Intermediate precision (%RSD)	0.2	0.2	0.4	

**Tab. 4.** Evaluation of Accuracy

**Amount Spiked**	**% Accuracy**							
		**Imp-A**	**Imp-B**	**Imp-C**	**Imp-D**	**Imp-E**	**Imp-F**	**Imp-G**
LOQ	% Recovery	92.9	101.1	96.0	96.0	94.4	101.8	100.1
	% RSD	4.2	3.0	6.0	4.0	0.0	3.4	6.3
50 %	% Recovery	95.7	100.9	105.1	101.7	102.0	93.9	101.1
	% RSD	0.37	1.27	0.22	0.39	0.39	1.83	1.2
100 %	% Recovery	101.9	102.4	98.5	99.3	101.3	93.2	97.5
	% RSD	0.40	0.70	0.30	0.20	0.29	0.12	0.5
150 %	% Recovery	106.0	102.2	98.2	99.6	102.1	94.6	98
	% RSD	0.84	0.20	0.20	0.26	0.60	0.31	0.5
200 %	% Recovery	98.7	102.1	99.2	98.8	101.3	98.7	97.1
	% RSD	0.54	0.10	0.15	0.23	0.25	0.58	0.2
300 %	% Recovery	98.1	101.0	99.6	99.7	100.5	99.1	98.5
	% RSD	0.10	0.10	0.15	0.20	0.16	0.25	0.1

% RSD values calculated with three sample recovery at each level.
